# Bilateral sequential accelerated theta-burst stimulation for treatment-resistant depression: an open-label randomized controlled trial

**DOI:** 10.1186/s12916-026-04799-8

**Published:** 2026-03-25

**Authors:** Xingxing Li, Enze Tang, Lingjiang Liu, Chang Yu, Kai Chen, Yongming Xu, Zhiwang Liu, Yuanyuan Liu, Mengqi Zhao, Shuangying Wang, Zichen Ding, Nan Jiang, Ti-Fei Yuan, Dongsheng Zhou

**Affiliations:** 1 Department of Psychiatry, Zhejiang Key Laboratory of Drug Addiction & Brain Health, Affiliated Kangning Hospital of Ningbo University (Ningbo Kangning Hospital), Ningbo, Zhejiang Province China; 2 Shanghai Key Laboratory of Psychotic Disorders, Brain Health Institute, National Center for Mental Disorders, Shanghai Mental Health Center, Shanghai Jiao Tong University School of Medicine and School of Psychology, Shanghai, China; 3 Co-innovation Center of Neuroregeneration, Nantong University, Nantong, Jiangsu China; 4 Ningbo Key Laboratory for Physical Diagnosis and Treatment of Mental and Psychological Disorders, Affiliated Kangning Hospital of Ningbo University, 1 Zhuangyu Road, Ningbo, Zhejiang China

**Keywords:** Treatment-resistant depression, Bilateral, Accelerated theta-burst stimulation, DLPFC

## Abstract

**Background:**

This study aims to evaluate the clinical effectiveness and feasibility of a novel bilateral sequential accelerated theta-burst stimulation (BS-aTBS) protocol in treatment-resistant depression (TRD) patients, compared to a standard intermittent TBS (a-iTBS) protocol.

**Methods:**

In this randomized controlled clinical trial, 94 TRD patients were randomly assigned to receive either BS-aTBS (*n* = 46) or a-iTBS (*n* = 48). Both groups delivered 10 daily TBS sessions (1800 pulses/session) for 5 consecutive days. The primary outcome was score on the 24-item Hamilton Depression Rating Scale (HDRS-24) after treatment, normalized to baseline (week 0).

**Results:**

Both BS-aTBS and a-iTBS protocols significantly reduced HDRS-24 scores by week 1 (65.52% vs. 54.32%, *p* = 0.008) and week 5 (77.10% vs. 67.77%, *p* = 0.006), and the BS-aTBS group showed significantly greater symptom improvement at both time points. Compared to a-iTBS, the BS-aTBS yielded higher response rates at week 1 (78.26% vs. 58.33%, *p* = 0.038) and week 5 (93.48% vs. 77.08%, *p* = 0.026), and elicited greater alleviation for anxiety and suicidal ideation at week 5. Moreover, clinical improvement on sleep quality and cognitive abilities was larger in the BS-aTBS group, and the effects on anhedonia and alexithymia were comparable between the two protocols. No serious adverse events were observed.

**Conclusions:**

The BS-aTBS protocol provided rapid and well-tolerated antidepressant effects in TRD patients lasting for 1 month, with additional benefits for anxiety and suicidal ideation. Considering its clinical advantages and shortened treatment time, the BS-aTBS may serve as a promising treatment protocol for TRD patients with high feasibility and efficiency.

**Trial registration:**

This trial was registered at Chinese Clinical Trial Registry (ChiCTR2400091459).

**Supplementary Information:**

The online version contains supplementary material available at 10.1186/s12916-026-04799-8.

## Background

Repetitive transcranial magnetic stimulation (rTMS), especially the theta-burst stimulation (TBS) scheme, is becoming a promising treatment option for treatment-resistant depression (TRD) [1, 2]. When compared to conventional rTMS protocols, advantages of TBS (e.g., intermittent TBS, iTBS; inhibitory continuous TBS, cTBS) [3, 4] include shorter treatment duration and long-lasting antidepressant efficacy [5]. Over recent years, the novel accelerated iTBS (a-iTBS) [6] and accelerated cTBS (a-cTBS) [7] protocols have been clinically validated as even more powerful for TRD patients [8], which reduced the treatment duration from several weeks to a few days.

However, common TBS protocols are administered unilaterally, which might inadequately modulate the network abnormality in bilateral hemispheres of TRD patients. Patients with depression display asymmetrical changes in the left and right hemispheres, characterized by the co-existence of hypo-activity of left dorsolateral prefrontal cortex (L-DLPFC) and hyper-activity of right DLPFC (R-DLPFC) [3, 9]. During treatment, left iTBS evoked excitatory effects by increasing the functional connectivity (FC) in the somatomotor network, subgenual anterior cingulate cortex (sgACC), and medial orbitofrontal cortex in the left hemisphere [10], while the right cTBS evoked inhibitory effects by decreasing the FC between the stimulated hemisphere and default mode network (DMN) [4, 11]. Alternatively, bilateral TBS stimulation may advantageously maximize the inter-hemisphere synergistic interactions, such as the FC between bilateral sgACC and DLPFC [12], thus hopefully decreasing individual variability in clinical responses and enhancing the antidepressant effectiveness [13, 14].

Another challenge for existing a-TBS schemes is the long daily treatment duration, such as 10 h per day. Hence, clinically ideal TBS protocols should further reduce daily treatment duration while maintaining the same amount of total pulses (90,000 pulses). Previous studies investigating the post-TBS cortical excitability levels indicated changes in motor evoked potentials (MEPs) lasting up to 60 min for iTBS or cTBS [15, 16]. Based on these findings, if a novel TBS scheme can appropriately set the ipsilateral between-session intervals as about 60 min and introduce bilateral stimulation with intervals as about 30 min, it will satisfactorily shorten the daily treatment duration to approximately 6 h while keeping the total number of pulses unchanged, thus balancing treatment efficiency and cortical plasticity effects.

In the present study, we took the initiative to design a novel bilateral sequential accelerated TBS (BS-aTBS) protocol, wherein L-DLPFC a-iTBS and R-DLPFC a-cTBS were sequentially implemented. We compared the effectiveness of the BS-aTBS scheme to improve clinical symptoms in TRD patients with the officially cleared L-DLPFC a-iTBS scheme. Specifically, both protocols delivered 10 daily sessions (1800 pulses/session) over 5 consecutive days. While the a-iTBS scheme took 10 h per day (50-min unilateral between-session interval), the BS-aTBS scheme took only 6 h per day (30-min bilateral between-session interval) with the same number of total pulses and recommended optimal unilateral between-session intervals [8]. We hypothesized that the BS-aTBS protocol can safely demonstrate greater improvements of depressive symptoms for TRD patients, and also satisfactorily reduce anxiety and suicidal ideation. The validated BS-aTBS protocol may warrant the development of more rapid and time-efficient neuromodulation strategies for TRD patients.

## Methods

### Study design

This clinical trial was registered in the Chinese Clinical Trial Registry (ChiCTR2400091459) before participant enrollment at http://www.chictr.org.cn. The study was approved by the Ethics Committee of Affiliated Kangning Hospital of Ningbo University (NBKNYY-2024-LC-62). The trial adhered to the Consolidated Standards of Reporting Trials guidelines and followed the ethical standards outlined in the Declaration of Helsinki. All participants signed written informed consent prior to any procedure.

In this open-label clinical trial, all participants were randomly assigned to the two treatment groups (i.e., BS-aTBS and a-iTBS groups). Patients received 10 daily treatment sessions for 5 consecutive days, with a 1-month follow-up period. Clinical assessments were administered at week 0 (w0, baseline), week 1 (w1, immediately after treatment), and week 5 (w5, 4 weeks after treatment).

### Participants

**Fig. 1:**
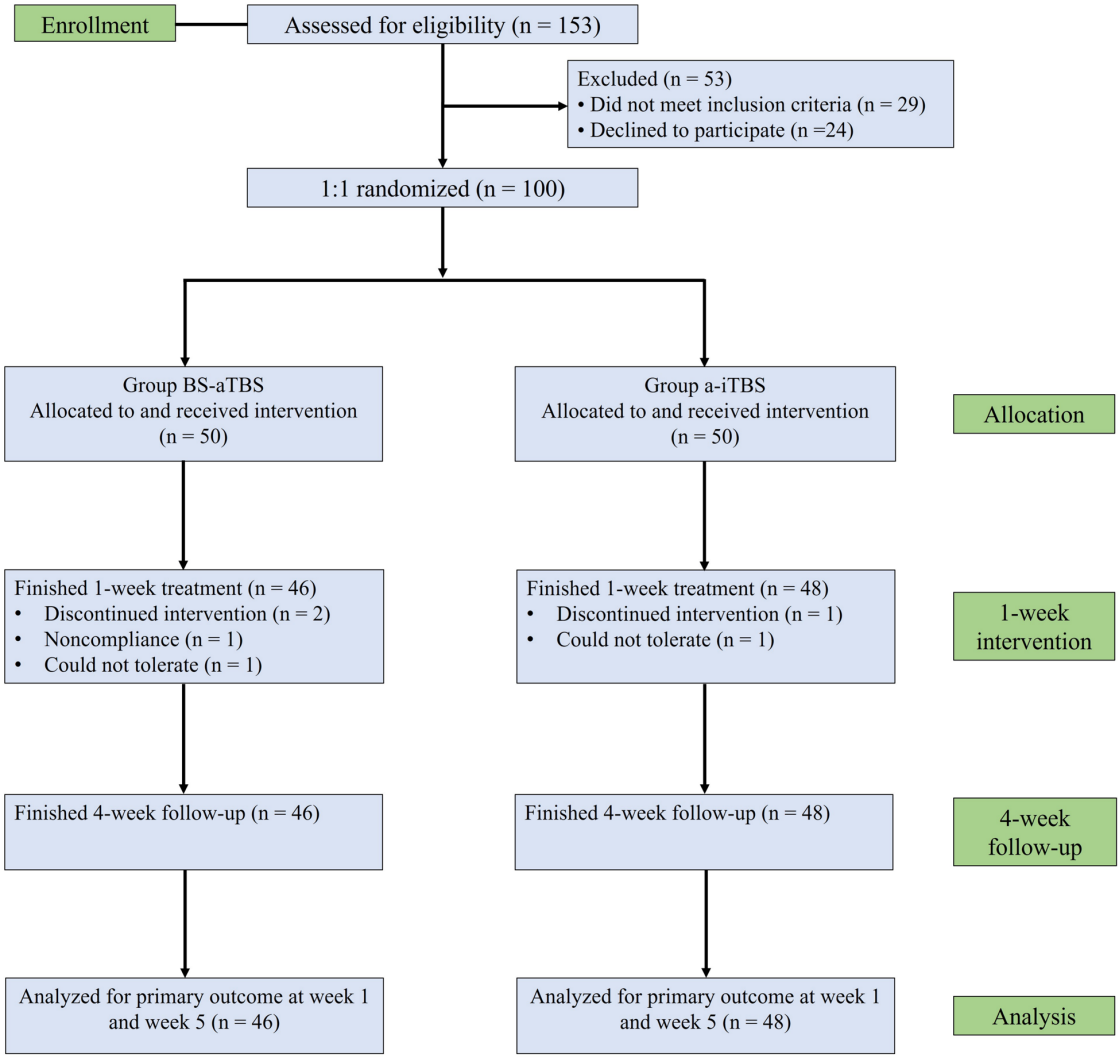
The CONSORT diagram of the clinical trial

Patients with TRD (*N* = 94) were recruited from a single center of Ningbo Kangning Hospital from November 2024 to August 2025, among whom 46 were randomly assigned to the BS-aTBS (a-iTBS + a-cTBS) group and 48 to the a-iTBS group (Fig. 1). All participants recognized themselves as Chinese and reported their biological sex at birth. The inclusion criteria included (1) clinical diagnosis of depression based on the DSM-5 criteria, which was confirmed independently by two experienced psychiatrists before any procedure; (2) moderate to severe level of therapeutic resistance according to the Maudsley staging method (MSM) and a score higher than 6 [17]; (3) a score of the Hamilton Depression Rating Scale-24 (HDRS-24) higher than 20; (4) age between 18 and 65 years; (5) voluntary participation in this study and able to provide written informed consent.

Exclusion criteria included (1) comorbidity with any psychiatric diagnosis other than depression (e.g., bipolar disorder, anxiety disorders and obsessive–compulsive disorder); (2) persistent depressive disorder/dysthymia, unspecified depressive disorder; (3) history of severe head injury, epilepsy, traumatic brain injury, or other neurological disorders; (4) receiving TMS, transcranial direct current stimulation (tDCS) or electroconvulsive therapy within the past 3 months; (5) inappropriate conditions for rTMS treatment, including metallic implants.

Criteria for discontinuation included (1) experiencing intolerable severe adverse effects and (2) voluntary requests to withdraw from the study.

### The BS-aTBS protocol

Prior to formal treatment, we used magnetic resonance imaging (MRI, Lianying Inc., Shanghai, China) method to acquire high-definition 3D T1-weighted images of each patient, and then imported them to the TMS navigation system (Weisi Inc., Nanjing, China) for precise and individualized localization of the stimulation targets (MagNeuro one). The MNI coordinates of the stimulation target at L-DLPFC and R-DLPFC were (− 44, 40, 29) and (44, 40, 29), respectively. [7, 18] Throughout this study, we used a TMS stimulator with figure-of-eight coil (Weisi Ltd, Nanjing, China). Drawing on existing accelerated TBS clinical trials [6, 19], the stimulation intensity was determined as 90% resting motor threshold (RMT) for a-iTBS and 80% for a-cTBS. The RMT was determined using electromyography (EMG) for all participants, which consistently recorded the abductor pollicis brevis muscle of the thumb contralateral to the stimulated M1 area. The treatment parameter settings refer to the RMT, and a single pulse mode self-driving coil is used to stimulate the M1 area of the treatment side to induce contralateral finger electromyographic signals (EMG > 50 μV is considered effective, 5 out of 10 times). Therefore, the RMT was determined separately for left and right hemispheres in the BS-aTBS group, and it was measured only for the left hemisphere in the a-iTBS group. Considering the noticeable variation of RMT in patients with TRD [20], the RMT was measured before each treatment session for all participants.

For the a-iTBS group, each 2-s iTBS train was comprised of 10 bursts of 3 pulses, wherein the frequency of the triplets (within-burst pulses) was 50 Hz and the between-burst frequency was 5 Hz. Every 2-s stimulation train was followed by an 8-s inter-train interval. For each treatment session, 1800 pulses were delivered over the stimulation duration of 10 min. There were 10 treatment sessions each day, with 50-min between-session intervals. In total, 18,000 pulses were administered per day, and the treatment duration lasted 5 consecutive days.

**Fig. 2:**
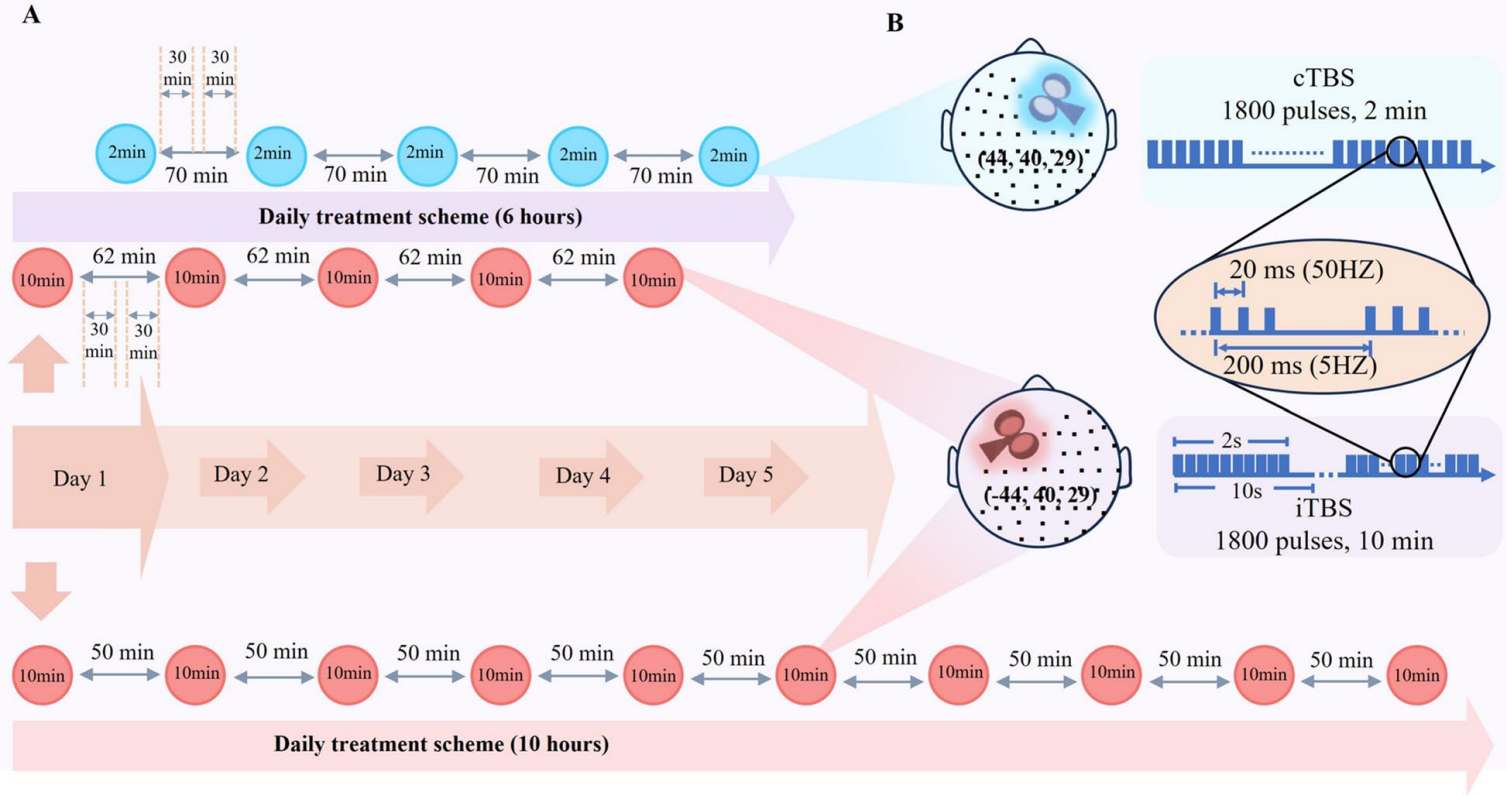
Stimulation protocols of two accelerated TBS. **A** The protocols of BS-aTBS (a-iTBS + a-cTBS) and a-iTBS. **B** Stimulation patterns and target sites for the sequential a-iTBS and a-cTBS

For the BS-aTBS group, we sequentially delivered the a-iTBS protocol at L-DLPFC first, and then, the a-cTBS protocol to the R-DLPFC. The parameters of the a-iTBS protocol here were identical to that of the a-iTBS group. For the a-cTBS protocol, the 50 Hz triplet were continuously delivered at 5 Hz, and 1800 pulses were delivered in 2 min for each session. For each day, 10 sessions were administered, including 5 a-iTBS and 5 a-cTBS sessions. The daily treatment duration was 6 h, as the bilateral inter-session interval was set as 30 min and the interval between two unilateral protocols was thus 1 h. In total, 18,000 pulses were administered per day, and the treatment duration lasted 5 consecutive days (see Fig. 2).

All participants were required to maintain a stable psychotropic medication regimen throughout the entire clinical trial, encompassing both intervention and follow-up periods. During the whole treatment, no adjustments in the type or dosage of psychotropic medications were made. In addition, no participants received any other NIBS treatment or psychotherapy/counseling throughout the whole trial.

### Clinical assessments

Clinical assessments were administered at w0 (baseline, 1 day before formal treatment), w1 (after treatment), and w5 (4 weeks after treatment). Depressive symptoms were assessed by HDRS-24. The primary clinical outcome was changes in the HDRS-24 score from baseline (w0) to w1 and w5, normalized to baseline. When participants’ total HDRS-24 score changed by 30% or more as compared from screening to the baseline assessment, they were ruled out of the research.

Secondary clinical assessments included anxiety symptoms measured by the Hamilton Anxiety Rating Scale (HAMA) and suicidal ideation measured by the Beck Scale for Suicidal Ideation (BSI). Secondary clinical outcomes included (1) daily changes of 6-item HDRS total score; (2) changes of the total score of HAMA and BSI; (3) response rate of HDRS-24, HAMA, and BSI; (4) remission rate of HDRS-24, HAMA, and BSI; and (5) adverse events. Other important clinical outcomes included Pittsburgh Sleep Quality Indexes (PSQI), Repeatable Battery for the Assessment of Neuropsychological Status (RBANS), Snaith-Hamilton Pleasure Scale (SHAPS), and Toronto Alexithymia Scale (TAS).

Before this clinical trial, three clinical assessors, who were qualified neuropsychiatrists blinded to participant groupings, received specialized training on all scales to ensure consistency and accuracy. We required an inter-rater correlation coefficient (ICC) no less than 0.8 for the training. Discrepancies were addressed through discussion, and additional patient cases were further independently assessed until the ICC for all scales was satisfied. For each participant, the same rater conducted all clinical assessments across different time points, which minimized within-subject measurement variability. During the trial, monthly meetings were held to calibrate the scoring criteria, discuss emerging questions, and reinforce adherence to rating guidelines. This rigorous procedure ensured unified standards and high reliability for assessments across evaluators.

### Randomization

Figure 1 presents the CONSORT flowchart of this clinical trial (Additional file 1: Table 1). For grouping, a researcher, who was not involved in any other procedure of the clinical trial, independently scripted a computer program and generated a random number (e.g., 1 or 2) table as the randomization sequence for all participants. Prior to the enrollment of the first patient, the group assignment information and corresponding participant ID were all stored in sealed and opaque envelopes. Once a patient was formally enrolled in this clinical trial, each patient was informed by the physician whether they would undergo unilateral or bilateral stimulation and the corresponding treatment duration. The operator was also made aware of the assigned group in order to administer the appropriate treatment protocol. Only the assessors and data analysts were blinded to the treatment allocation.

### Statistical analysis

All statistical analyses were conducted using SPSS version 27.0. We conducted power analyses using G*Power to determine the minimum sample size for the estimation of differences in changes of HDRS-24 score between the two groups. We determined to use the between-factor model of repeated-measures analysis of variance (ANOVA) and assumed a medium effect size (0.3). To achieve 90% statistical power (*α* = 0.05), 90 participants were the minimum required. With an assumed 10% drop-out rate for the treatment, we originally enrolled 100 patients for randomized grouping.

The primary outcome measure was the percent reduction of HDRS-24 score at w1 and w5 after treatment, which was calculated as [[(HDRS-24_post_ − HDRS-24_pre_)/HDRS-24_pre_] * 100%]. For all analyses of suicidal ideation, only patients with a BSI score ≥ 9 was included, so that the data from a subset of 29 and 31 participants were used in the BS-aTBS and a-iTBS groups, respectively. Percentage changes in HAMA and BSI scores were used as secondary outcomes. Clinical response for depression, anxiety, and suicidal ideation was defined as a reduction ≥ 50% from baseline for the HDRS-24, HAMA, and BSI scores. Remission was defined as a HDRS-24 score < 9 for depression, a HAMA score < 8 for anxiety, and a BSI score < 9 for suicidal ideation. Normality was assessed using the Kolmogorov–Smirnov test, while sphericity and homogeneity of variance were tested using Mauchly’s and Levene’s tests, respectively. Demographic and clinical variables between groups were analyzed using ANOVA and chi-square (*χ*^2^) tests.

This longitudinal study analyzed the effectiveness of two different interventions (a-iTBS and BS-aTBS) on depressive symptom, anxiety, and suicidal ideation. Using repeated-measures ANOVA, three time points (w0, w1, w5) were set as within-group repeated measures, and the intervention group was set as the between-group variable. If the interaction between time and group was statistically significant, post hoc pairwise comparisons were conducted, and the *p*-values were corrected by the Bonferroni method. At each follow-up time point, the response and remission rates of the two groups were compared by chi-square (*χ*^2^) tests. To analyze changes in the score of clinical assessments, we used independent *t*-tests, with two-tailed *p* ≤ 0.05 considered as statistically significant.

## Results

### Clinical characteristics

In total, 153 patients from mainland China were screened for this study (Fig. 1). However, 29 patients did not meet the inclusion criteria, and 24 patients declined to participate. The remaining 100 patients were equally and randomly assigned to the BS-aTBS and a-iTBS groups. During the 5-day treatment, 4 patients (2 for discontinued intervention due to voluntary requests; 1 for noncompliance; 1 for intolerance) in the BS-aTBS group and 2 patients (1 for discontinued intervention due to voluntary requests; 1 for intolerance) in the a-iTBS group withdrew from this study. No patients were lost during the 1-month follow-up. Hence, data of 46 patients in the BS-aTBS group and 48 patients in the a-iTBS group were analyzed. In the BS-aTBS group, the mean MSM score was 10.70 ± 2.17, whereas in the a-iTBS group, the mean MSM score was 10.33 ± 2.36. There were no significant differences in demographic or clinical characteristics between the two groups at baseline (Table 1).

**Table 1:** Baseline demographic and clinical characteristics

Characteristic	Treatment group		*F* or *χ*^2^ (*p* value)
	BS-aTBS (*n* = 46)	A-iTBS (*n* = 48)	
Age (y)	31.54 ± 16.15	32.81 ± 16.10	0.145 (0.704)
Gender (M/F)	15:31	14:34	0.130 (0.718)
Years of education (y)	12.04 ± 3.38	13.60 ± 5.57	2.673 (0.105)
Episode duration (y)	4.59 ± 6.52	4.98 ± 5.11	0.108 (0.743)
BMI	21.40 ± 3.97	21.61 ± 3.41	0.074 (0.786)
Antidepressants type			0.197 (0.978)
Escitalopram	27	29	
Fluoxetine	7	6	
Sertraline	5	6	
Paroxetine	7	7	
Baseline clinical measures			
MSM score	10.70 ± 2.17	10.33 ± 2.36	0.601 (0.440)
HDRS-24, 24-item	29.33 ± 5.22	29.19 ± 5.37	0.016 (0.899)
HDRS-24, 6-item	11.61 ± 3.04	11.69 ± 3.30	0.014 (0.905)
HAMA score	20.65 ± 7.98	19.96 ± 5.73	0.236 (0.628)
BSI total score	17.83 ± 6.57	17.29 ± 4.91	0.130 (0.720)
PSQI	12.26 ± 3.21	12.46 ± 3.29	0.087 (0.769)
RBANS	92.20 ± 15.91	95.71 ± 11.79	1.487 (0.226)
SHAPS	31.74 ± 6.52	32.71 ± 6.48	0.522 (0.472)
TAS	61.39 ± 9.88	59.77 ± 12.39	0.489 (0.486)

The data are presented as mean ± standard deviation (SD)

*Abbreviation*: *A-iTBS*, accelerate intermittent theta burst stimulation; *BMI*, body mass index; *BS-aTBS*, bilateral sequential accelerated theta burst stimulation; *BSI*, Beck Scale for Suicide Ideation; *HAMA*, Hamilton Anxiety Scale; *HDRS-24*, Hamilton Depression Rating Scale-24; *MSM*, Maudsley staging method; *PSQI*, Pittsburgh Sleep Quality Index; *RBANS*, Repeatable Battery for the Assessment of Neuropsychological Status; *SHAPS*, Snaith-Hamilton Pleasure Scale; *TAS*, Toronto Alexithymia Scale

### Primary outcome

**Fig. 3:**
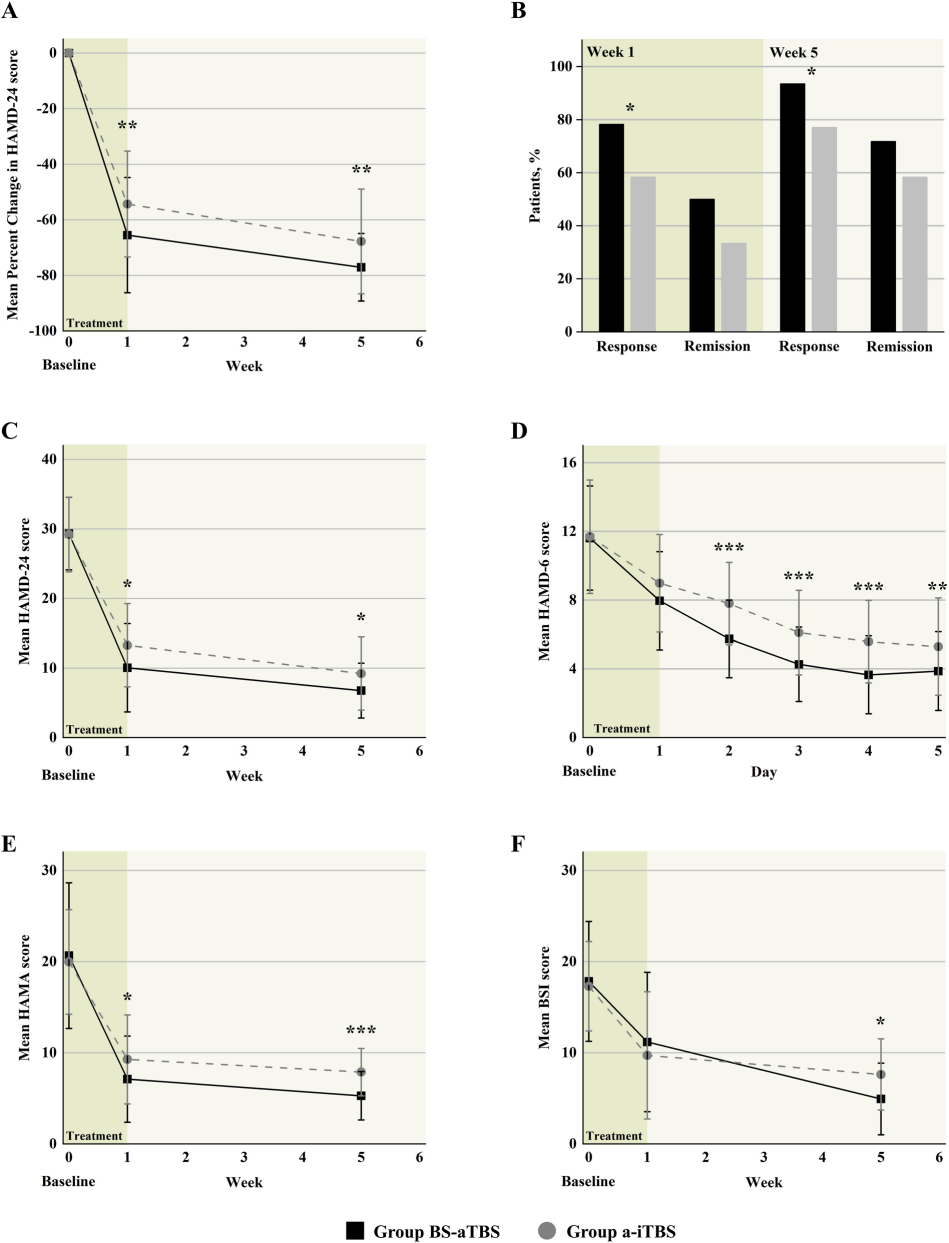
Clinical outcomes. **A** Reduction in HDRS-24 score. **B** The remission rate and response rate of the two accelerated TBS groups. **C** Changes in HDRS-24 score. **D** Changes in HDRS-6 score. **E** Changes in HAMA score. **F** Changes in BSI score. *p < 0.05, **p < 0.01, ***p < 0.001

The primary outcome was the reduction of HDRS-24 score at w1 and w5 after treatment (Fig. 3). To compare the reduction in HDRS-24 scores, the mixed-design ANOVA analyses revealed significant main effect of group (*F*_1,92_ = 5.513, *p* = 0.021, *η*^2^ = 0.057) and time (*F*_2,184_ = 550.747, *p* < 0.001, *η*^2^ = 0.857), with a significant interaction effect (*F*_2,184_ = 3.306, *p* = 0.039, *η*^2^ = 0.035). The time main effect indicated that HDRS-24 scores (Table 2) at all follow-up time points were significantly decreased compared to baseline (all *p*s < 0.001). Post hoc analyses on the interaction effect indicated greater improvement of depressive symptoms in the BS-aTBS group at the two follow-up time points (w1: *F*_1,92_ = 7.460, *p* = 0.008, *η*^2^ = 0.075; w5: *F*_1,92_ = 8.053, *p* = 0.006, *η*^2^ = 0.080), compared to the a-iTBS group (BS-aTBS percent change at weeks 1, 5 = 65.52%, 77.10%; a-iTBS percent change at weeks 1, 5 = 54.32%, 67.77%).

**Table 2:** Symptom scale scores at baseline, week 1, and week 5

	Baseline	Week 1	Week 5	Group F (*p* value)	Time F (*p* value)	Group * Time F (*p* value)
HDRS-24 scores				5.513 (0.021)	550.747 (< 0.001)	3.306 (0.039)
BS-aTBS (*n* = 46)	29.33 ± 5.22	10.04 ± 6.34	6.76 ± 3.94			
A-iTBS (*n* = 48)	29.19 ± 5.37	13.27 ± 5.98	9.21 ± 5.25			
HAMA scores				3.050 (0.084)	297.400 (< 0.001)	4.213 (0.016)
BS-aTBS (*n* = 46)	20.65 ± 7.98	7.11 ± 4.73	5.28 ± 2.65			
A-iTBS (*n* = 48)	19.96 ± 5.73	9.27 ± 4.88	7.88 ± 2.60			
BSI scores				0.032 (0.858)	129.318 (< 0.001)	4.697 (0.011)
BS-aTBS (*n* = 29)	17.83 ± 6.57	11.17 ± 7.64	4.93 ± 3.93			
A-iTBS (*n* = 31)	17.29 ± 4.91	9.71 ± 6.97	7.61 ± 3.91			

The data are presented as mean ± standard deviation (SD)

*Abbreviation*: *BS-aTBS*, bilateral sequential accelerated theta burst stimulation; *A-iTBS*, accelerate intermittent theta burst stimulation; *HDRS-24*, Hamilton Depression Rating Scale-24; *HAMA*, Hamilton Anxiety Rating Scale; *BSI*, Beck Scale for Suicidal Ideation

### Secondary outcomes

Statistical details for secondary outcomes are provided (Table 2; Additional file 1: Table 2). For daily changes in 6-item HDRS score (Additional file 1: Table 3), there was a significant interaction effect between time and group (*F*_5,460_ = 4.478, *p* < 0.001, *η*^2^ = 0.046). From the second day to the end of treatment, patients in the BS-aTBS group had significantly lower scores than in the a-iTBS group (all *p*s < 0.05) (Fig. 3). Compared to a-iTBS, the BS-aTBS protocol decreased the HAMA (*p* = 0.127) and BSI (*p* = 0.449) scores comparably at w1, but it showed significantly larger alleviation of anxiety (*p* < 0.001) and suicidal ideation (*p* = 0.002) at w5.

The response rates of depression in the BS-aTBS group (w1: 78.26%, w5: 93.48%) were significantly higher than the a-iTBS group (w1: 58.33%, w5: 77.08%) at both w1 (*p* = 0.038) and w5 (*p* = 0.026). For anxiety symptoms and suicidal ideation, the response rates in the BS-aTBS group (91.30% and 89.66%, respectively) were significantly higher than the a-iTBS group (75.00% and 61.29%, respectively) at w5 (anxiety: *p* = 0.035; suicidal ideation: *p* = 0.011). The remission rates of depressive symptoms were comparable at both follow-up time points (w1: *p* = 0.101; w5: *p* = 0.173) between the two groups (BS-aTBS: 50.00% at w1 and 71.74% at w5; a-iTBS: 33.33% at w1 and 58.33% at w5). However, the BS-aTBS group showed higher remission rates of anxiety scores (w1: 60.87% vs. 39.58%, *p* = 0.039; w5: 84.78% vs. 50.00%, *p* < 0.001) and suicidal ideation (w5: 82.76% vs. 51.61%, *p* = 0.011) than the a-iTBS group.

The treatment effects of BS-aTBS and a-iTBS on other important clinical outcomes were also evaluated (Additional file 1: Table 4). Specifically, BS-aTBS showed greater improvement on sleep quality (*F*_2,184_ = 6.394, *p* = 0.002, *η*^2^ = 0.065) and cognitive abilities (*F*_1.404,129.155_ = 5.718, *p* = 0.010, *η*^2^ = 0.059). As measured by PSQI (w1: *p* = 0.232; w5: *p* = 0.001) and RBANS (w1: *p* = 0.034; w5: *p* = 0.008), discrepancy in clinical outcomes between the two protocols occurred at different time points. In addition, both treatment protocols significantly improved SHAPS and TAS scores, and their clinical effectiveness was comparable throughout (SHAPS: *F*_1.534,141.109_ = 1.200, *p* = 0.295; TAS: *F*_1.435,132.056_ = 2.169, *p* = 0.133).

### Safety

No serious adverse events occurred throughout the study (Additional file 1: Table 5). Two groups reported similar side effects, with the most common being fatigue, headache, and dizziness. Other sporadically reported side effects included insomnia, abnormal sensations, nausea, and tinnitus. All reported side effects were addressed spontaneously on completion of treatment, without the need for additional medical intervention. In general, patients tolerated the treatment well, without seizures or manic symptoms.

## Discussion

For the first time, this study compared the clinical effectiveness of a bilateral sequential accelerated TBS protocol with the FDC-cleared a-iTBS protocol in patients with TRD. Compared to the a-iTBS protocol, we observed a greater reduction of depressive severity in the BS-aTBS group immediately after the 5-day treatment, and its advantages lasted during the 4-week follow-up period. The response rate of depressive symptoms in the BS-aTBS group was higher than the a-iTBS group at all follow-up points, but their remission rates were comparable throughout. In addition, we also observed delayed advantages of symptom improvement, response rate, and remission rate for anxiety and suicidal ideation in the BS-aTBS group, especially at week 5. Regarding other clinical measures, improvement on sleep quality and cognitive abilities was larger in the BS-aTBS group, and the effects on anhedonia and alexithymia were comparable between the two protocols. As such, the BS-aTBS protocol not only shortened the daily treatment duration from 10 to 6 h without serious adverse events but also demonstrated greater improvement of depressive symptoms, anxiety, and suicidal ideation. Therefore, these findings suggest that the BS-aTBS protocol may be a promising time-efficient treatment for patients with TRD.

Both protocols substantially decreased the HDRS-24 scores as fast as 5 days (w1), but the BS-aTBS protocol showed higher reduction (65.52%) and response rate (78.26%) than the a-iTBS protocol (reduction: 54.32%; response rate: 58.33%). According to the core 6-item HDRS scale, the advantages of the BS-aTBS protocol became apparent by the second day of treatment, further demonstrating its rapid effects on depression alleviation. Current findings corroborated previous clinical trials reporting greater symptom improvement of bilateral sequential TBS schemes [5] than unilateral TBS schemes, especially for TRD patients [3, 21]. While the precise neurobiological mechanisms were not explicitly investigated, the observed effects might be interpreted through the lens of the Bienenstock, Cooper, and Munro (BCM) theory of synaptic homeostasis [22]. Hypothetically, the preceding a-iTBS session may increase cortical excitability in the L-DLPFC and project the changes to broader brain regions, including the R-DLPFC [23], which potentially lowers the threshold for long-term depression-like effects (LTD) induced by the subsequent a-cTBS. In other words, the sensitivity of TRD patients to the a-cTBS could be upregulated by a priming a-iTBS session. Regarding the maintained advantages of BS-aTBS over a month follow-up period, it may reflect gradually unfolding modulation of neurotrophic factors and signaling pathways [24].

Interestingly, the two TBS protocols had significant differences only in response rates, rather than the remission rates of depression. By definition, response rate was measured by within-patient self-comparison, referred to as a reduction ≥ 50% from baseline, while the remission rate was determined by a cut-off score of 9 for the HDRS-24. Existing clinical trials enrolling depressive patients have mostly reported higher response rates than the other [25]. Since response rate was calculated with a consideration of the symptomatic severity at baseline, it could more sensitively detect changes in depressive symptoms than remission rate. In support of this view, recent studies have identified much more candidate predictors of remission rate (e.g., demographic information, comorbidity and other clinical profiles) than the response rate (e.g., 5-HT transporter polymorphisms, and depressive symptomatology) for depressive patients [26], suggesting that remission rate might be more difficult and complex to be modulated by TBS parameters only. As a result, for the core symptom of depression, the BS-aTBS protocol showed significant improvement in the sensitive and straightforward metric of response rate, but did not show significant effects on remission rate. Although this study observed favorable outcomes of BS-aTBS for the treatment of TRD, the etiology and neurobiological mechanisms of TRD are highly complex, so that the achievement of full remission may require adjunctive psychosocial or pharmacological interventions.

The BS-aTBS protocol also showed delayed advantages to alleviate anxiety and suicidal ideation at week 5, compared to the a-iTBS scheme. Although the current study did not explicitly investigate the precise mechanisms, we hypothesize that the temporal dissociation of the effectiveness on different symptom dimensions may reflect differential circuit-level modulation. It could be that rapid improvement in depressive symptoms might stem from the direct and early engagement of mood regulatory networks, including the DLPFC and cingulate-striatal circuits [27, 28]. Afterwards, the more gradual and downstream modulation of amygdala-prefrontal pathways [29] or the reorganization of large-scale network connectivity functioned to alleviate anxiety and suicidal ideation [30]. This implies that cortical excitability may be modulated quickly, while the top-down regulation of subcortical regions associated with anxiety and suicide impulse control could involve a prolonged neuroplastic process. Alternatively, such effects might be associated with the accumulation of self-feelings for changes in patients themselves and to the sensitivity of the assessment scales. Previous studies identified more pronounced effects of TBS protocols at the last follow-up assessment [31] contended that longer follow-up duration (e.g., 6 weeks) might capture patients’ delayed responses to treatment [32, 33], as personal feelings for anxiety or suicidal ideation might continue decreasing over time after the treatment. Also, longer scales measuring these clinical symptoms seemed less sensitive than scales with fewer items to detect short-term changes induced by antidepressant treatment [34, 35], so the reflection of discrepancy in anxiety and suicidal ideation changes between the two protocols required a longer follow-up period (w5).

Some factors might be crucially relevant with the greater improvement of clinical symptoms of the BS-aTBS protocol. First, it may result from both shortened daily treatment duration and maintained 60-min unilateral intersession interval. Compared to the a-iTBS protocol, we decreased the daily treatment duration from 10 to 6 h, wherein the bilateral interval was 30 min and the unilateral interval was 60 min. An appropriately shorter treatment duration is indeed important, as a prolongation of stimulation duration for TBS schemes may lead to diminished effectiveness or even reversed neuroplastic aftereffects [36]. In the meanwhile, the unilateral intersession interval is suggested to be set as 50–90 min, [37, 38] which can maximize clinical outcomes through additive synaptic modification and metaplasticity.

In addition, the sequence of a-iTBS followed by a-cTBS could be an important influential factor. Previous studies have considered stimulation at L-DLPFC as a “top-down” augmentation, so that an a-iTBS session will possibly engender a cascade of modulation effects in the R-DLPFC [3], wherein the abnormally altered frontal-limbic areas [39] and the asymmetrical frontal cortical activity [40] in patients with depression can be restored. Hence, in consistent with an open-label clinical trial [14], we have determined to sequentially implement a-iTBS and then a-cTBS to maximize the interactive synergy. However, the BMC theory [22] did not specifically point out the lateral differences in modifying the threshold for synaptic plasticity (i.e., long-term potentiation and depression), it thus remains unknown whether an a-cTBS + a-iTBS sequence would have different clinical effectiveness compared to our current design. Future studies are suggested to continue investigating whether the reversed a-cTBS + a-iTBS sequence would significantly change the clinical outcomes.

Beyond stimulation parameters, subjective expectancy also represents a critical factor. Given the open-label design and the substantial disparity in daily treatment duration (10 h vs. 6 h), participants receiving the BS-aTBS protocol likely experienced lower treatment burden, potentially developing more favorable expectations of clinical benefit [41]. Previous evidence showed that enhanced expectancy could bolster engagement motivation, increase acceptability, and mitigate fatigue, thereby potentiating symptom improvement through non-specific mechanisms [42, 43]. Consequently, the observed between-group differences in symptom improvement could be a combination of stimulation effects and expectancy-related influences, rather than being attributable solely to protocol-specific biological mechanisms. Future investigations involving more sophisticated designs are warranted to disentangle the specific neurophysiological effects of stimulation from the non-specific contributions of treatment duration and expectancy.

Our study introduces significant novelties to the field of accelerated neuromodulation. While prior work has established the feasibility of BS-aTBS in TRD patients, generalization has been questioned by certain limitations, including the insufficient control for psychiatric comorbidities and limited follow-up [44, 45]. Distinguishing itself from studies comparing BS-aTBS protocols with high-frequency rTMS [45, 46] or no other protocols [44, 47, 48], this is the first RCT to compare the antidepressant effectiveness between BS-aTBS and the FDA-cleared a-iTBS scheme over a 1-month follow-up period, with a more comprehensive examination of depression, anxiety, and suicidal ideation symptoms. Additionally, by using a sequential stimulation design, we decreased the safety risks associated with simultaneous delivery while maximizing theoretical inter-hemispheric synergistic effects. Our findings indicate greater symptom improvement of BS-aTBS in TRD patients compared to the unilateral accelerated protocol, supporting further investigation of its clinical translation.

This study has several limitations. First, this was a single-center clinical trial that directly compared the effectiveness of two TBS protocols (i.e., BS-aTBS and a-iTBS) to improve symptoms without including a sham group. Hence, the homogeneity of demographic and clinical characteristics in the single-center sample might generate critical concerns for the validity of current findings. Future investigations should prioritize large-scale, multi-center, randomized, sham-controlled trials to validate and generalize current findings using sham-controlled groups and diversified TRD samples (e.g., various age and ethnic populations). Moreover, these trials would also benefit from incorporating biomarker-based stratification to identify the sub-populations preferentially respond to the BS-aTBS protocol. Second, the present study primarily evaluated overall clinical effectiveness under pragmatic real-world conditions, rather than isolating specific neurophysiological mechanisms. Future investigations should involve rigorous designs to dissect specific contributions of key parameters, such as treatment duration, aTBS sequence and expectancy effects, thereby isolating the biological mechanisms attributable to the stimulation. Third, this trial only involved a relatively short follow-up period (4 weeks), which precluded conclusions regarding long-term stability, relapse prevention, and delayed adverse effects associated with the BS-aTBS protocol. In this case, future studies with extended follow-up period (e.g., 3 to 6 months) are needed to assess the durability of clinical improvements, supplementing important information for clinical guidance. Fourth, substantial differences in daily treatment duration (6 h versus 10 h) and the number of stimulation sites between study arms could have impacted subjective experience (e.g., fatigue, irritability, concentration) and expectancy of patients, potentially modulating clinical symptom changes.

## Conclusions

In conclusion, this study provides evidence that the BS-aTBS protocol may achieve rapid improvement of depressive symptoms in TRD patients lasting for one month, with 6-h daily treatment duration. In addition, the BS-aTBS protocol also showed delayed clinical benefits on the alleviation of anxiety symptoms, suicidal ideation, sleep quality, and cognitive abilities. These findings suggest that the BS-aTBS protocol may be a promising, time-efficient and clinically feasible treatment option for TRD. Regarding clinical implementation, the BS-aTBS protocol may have potential for integration into routine workflows for TRD patients in both inpatient and outpatient settings, which prioritizes rapid symptom relief within a highly compressed treatment course.

## Supplementary Information

Additional file 1: Tables 1–5. Table 1 – [CONSORT checklist]. Table 2 – [Primary and secondary indicators by two TBS intervention groups]. Table 3 – [Raw scores on the 6-item HDRS]. Table 4 – [Supplementary clinical indicators by two TBS intervention groups]. Table 5 – [Tolerability].

## Data Availability

The data that support the findings of this study are available on reasonable request from the corresponding author.

## Abbreviations

TRD: Treatment-resistant depression
rTMS: Repetitive transcranial magnetic stimulation
TBS: Theta-burst stimulation
a-iTBS: Accelerated intermittent theta-burst stimulation
a-cTBS: Accelerated continuous theta-burst stimulation
BS-aTBS: Bilateral sequential accelerated theta-burst stimulation
L-DLPFC: Left dorsolateral prefrontal cortex
R-DLPFC: Right dorsolateral prefrontal cortex
RMT: Resting motor threshold
MEP: Motor evoked potential
MSM: Maudsley staging method
BSI: Beck Scale for Suicidal Ideation
HAMA: Hamilton Anxiety Rating Scale
HDRS-24: Hamilton Depression Rating Scale-24
